# Cognitive Influences in Parkinson's Disease Patients and Their Caregivers: Perspectives From an Australian Cohort

**DOI:** 10.3389/fneur.2021.673816

**Published:** 2021-11-15

**Authors:** Michal Lubomski, Ryan L. Davis, Carolyn M. Sue

**Affiliations:** ^1^Department of Neurology, Royal North Shore Hospital, Northern Sydney Local Health District, St Leonards, NSW, Australia; ^2^Department of Neurogenetics, Kolling Institute, Faculty of Medicine and Health, University of Sydney, Northern Sydney Local Health District, St Leonards, NSW, Australia; ^3^School of Medicine, The University of Notre Dame Australia, Sydney, NSW, Australia

**Keywords:** Parkinson's disease, cognition, caregiver, cognitive impairment, dementia

## Abstract

**Objectives:** Cognitive impairment impacts negatively on Parkinson's disease (PD) patient and caregiver quality of life (QoL). We examined cognitive impairment in PD patients and their caregivers to determine if caregiver cognition affected their PD relative.

**Methods:** Validated cognition and clinical outcome measures were assessed in 103 PD patients and 81 caregivers.

**Results:** PD patients showed more cognitive impairment than their carers, with 48.6% having possible Mild Cognitive Impairment (MCI) and 16.5% having PD dementia. Increasing age, male gender, lower education level, various non-motor symptoms and certain therapies, associated with poorer cognition in PD. Eighteen and a half percent of caregivers were found to have MCI, in association with a lower physical and mental QoL. This reflected in lower QoL and mood for the respective PD patients.

**Conclusion:** Impaired cognition and QoL in caregivers was associated with decreased QoL and mood for respective PD patients, suggesting MCI in caregivers is an important consideration for the management of PD.

## Introduction

Parkinson's disease (PD) is a progressive multisystem disorder that contributes to significant morbidity, healthcare and caregiver burden (1, 2). Cognitive impairment can be present in as many as 30% of PD patients at disease onset, progressing to PD Dementia (PDD) in up to 80% of patients with advanced disease (3). The concept of mild cognitive impairment (MCI) is an intermediate clinical state between normal cognitive ageing and dementia (4). MCI represents an important window in time where patients are still functional in their day to day life but are at a higher risk of developing PDD (5). Despite a growth in literature evaluating cognitive changes in PD patients (3, 6–8), there is a lack of understanding of the differences in cognition between PD patients and their caregivers (9, 10).

It has been suggested that PD caregivers experience a higher burden of care leading to reduced quality of life (QoL) with the onset of dementia in their PD relative (11), influencing PD management and delaying formal care or nursing home placement (12). As there is limited evidence around the influence of PD caregivers with cognitive impairment and their capacity to care for a PD patient, understanding these aspects could inform best clinical practise to improve caregiver well-being for better support and care of PD relatives.

Individuals with PDD commonly report deficits in attention, language, memory and visuospatial orientation (13). Additionally, deficits in executive functioning affect daily tasks, such as driving and medication adherence, whilst other individuals show a reduced awareness of their executive deficits and overestimate their capabilities (13). These impacts are likely to negatively influence caregiver QoL and further add to caregiver burden. Several prior studies have evaluated PD-related risk factors for cognitive impairment, highlighting potentially modifiable specific risk interventions (14, 15). Equally, non-modifiable factors, such as age, age at diagnosis, rigid-akinetic phenotype, physical impairment, impairment of semantic fluency, genetic factors, low education level and postural instability have also been proposed as important risk factors for PDD (15).

We hypothesised that a greater degree of cognitive impairment in PD patients would result in perceptibly lower QoL and enhancement of non-motor symptoms (NMS). Additionally, we hypothesised that the presence of cognitive impairment in PD caregivers results in a worsening of their QoL, which could potentially impact on their ability to effectively care for their affected PD relative. The study presented herein provides new insight into PD caregiver cognitive impairment and the potential impact on PD patient care and well-being.

## Methods

### Study Settings and Subjects

Subjects were recruited between June 2018–2019 from the movement disorder and neurology clinics at Royal North Shore Hospital, Sydney, Australia, as reported in our previous study (16). Consecutive PD patients were recruited if they were >18 years of age, and had a clinical diagnosis of idiopathic PD according to the UK Parkinson's Disease Society Brain Bank Diagnostic Criteria (17). Caregivers were recruited if they were >18 years of age, they exhibited no clinical indication of PD, they had no known diagnosis of a neurodegenerative disorder, and were a spouse, sibling or child residing in the same abode. Caregivers were chosen as the comparator group due to their availability and relative matched age to PD patients. Exclusion criteria included secondary Parkinsonism and medical or surgical disorders preventing completion of questionnaires. Patients were required to read and complete the questionnaires in English language, which resulted in the exclusion of 1 PD participant. Existing cognitive impairment, motor symptom limitation, mental illness or traumatic brain injury were not considered exclusion criteria. All PD patients and caregivers were specifically asked to complete their own questionnaires.

### Data Collection

Data was collected from validated self-administered questionnaires, in addition to data on socio-demographic, lifestyle and clinical management factors (16). Both PD patients and caregivers completed the same questionnaires described below, unless the questionnaire was PD-specific (indicated by ^*^). The Montreal Cognitive Assessment (MoCA) (18) has been validated as the most appropriate screening instrument for the detection of MCI and PDD (5). It is also endorsed by the International Parkinson and Movement Disorder Society (MDS) Task Force for the diagnosis of PD-MCI as being most suitable for demonstrating global cognitive deficits in a clinical setting (19). The MoCA was utilised to screen for MCI (score <26/30) (5, 18, 20) and PDD (score of <21/30 and loss of one or more instrumental activity of daily living) (20). Level 1 MDS criteria were used to assess for possible MCI (21). One consultant neurologist investigator (ML) conducted all MoCA assessments with PD patients and caregivers separately, to minimise bias. The Short Form Health Survey (SF-36) (22) was used to assess QoL for both PD patients and their caregivers to examine perceived health status. Two aggregate summary scores, the Physical Component Summary (PCS) and the Mental Component Summary (MCS) were derived from the eight concepts. Each scale was scored from 0 (most disability) to 100 (least disability). PD patients also completed the PDQ-39 Questionnaire^*^ (23), a validated measure of QoL in PD. Clinical depression was determined by the Beck Depression Inventory (BDI), with scores ranging 0–63 (24). Depression was diagnosed in accordance with validated BDI cut-off criteria for PD patients (>13) and caregivers (>9) (25, 26).

A number of other questionnaires were used to assess non-motor symptoms in the PD cohort (27). Constipation severity was evaluated by the Rome-IV criteria (28) and the Cleveland Constipation Score (CCS) (29). Physical activity was assessed by the International Physical Activity Questionnaire (IPAQ) (30) and NMS were assessed by the Non-Motor Symptoms Scale^*^ (NMSS), scored between 0 (least affected) to 243 (most affected) (31). Clinical motor assessments were performed by a neurologist during the patient's ‘on’ state, as a measure of the existing motor function, in accordance with the International Parkinson and Movement Disorder Society—Unified Parkinson's Disease Rating Scale—Part III (MDS-UPDRS III) criteria^*^ (32). PD phenotype was determined from MDS guidelines (33). PD medications were compared following standard methods for calculating daily levodopa equivalent dose (LED)^*^ (34), whilst Impulse Control Disorder (ICD)^*^ was defined according to established diagnostic criteria (35).

### Statistical Analysis

Normal distribution of all data was confirmed using the Shapiro-Wilk test. Two-sample, independent *t*-tests were used to analyse differences between the groups for continuous variables. Chi-squared tests were used to compare differences between categorical variables. Logistic and linear regression models were constructed to evaluate differences in the prevalence of cognitive features between the PD and caregiver groups, as well as within the PD cohort, after controlling for demographic and clinical variables. Pearson correlations were used to evaluate relationships between clinically relevant variables. *p* < 0.05 was set as the level of statistical significance. Data analysis was performed using SPSS, version 26 (SPSS Inc, Chicago, IL, USA).

## Results

### Demographic Characteristics

Demographic information pertaining to the cohort studied here has been reported previously (16). In summary, 103 PD patients, mean age 67.1 years [Standard Deviation (SD 12.2)], 56.3% male and 81 caregivers, mean age 62.4 years (SD 15.6), 32.1% male were recruited (Table 1). Within the PD cohort, the mean age of symptom onset was 58.8 years (SD 13.6) and mean duration of disease was 9.2 years (SD 6.5). Half of the PD patients reported a late disease onset (>60 years), whilst 11% had early onset (<40 years) (Table 2). Of the NMS, half of the PD patients reported Rapid Eye Movement Sleep Behaviour Disorder (RBD), three-quarters reported hyposmia and one fifth identified having an impulse control disorder (ICD). In terms of risk factors for cognitive impairment, 18.4% had a family history of PD, 10.7% reported a significant history of head trauma, and 2.9% had previously used neuroleptic medication. Approximately 5% of the PD cohort was treatment naïve, whilst those on treatment regimens had a mean daily LED of 834.8 mg (SD 527.3). The mean MDS-UPDRS III score was 32.9 (SD 17.9). Utilisation of standard and device assisted therapies, the frequency and severity of gastrointestinal symptoms, chronic pain, physical activity and NMS in the PD cohort are further outlined in Table 2.

**Table 1:** Cohort demographic and clinical characteristics.

	**Parkinson's disease**	**Caregivers**	**Test statistic**	***p*-value**
Number of patients (*n* = )	**103**	**81**		
Mean age (years) (SD, range)^*^	67.1 (12.2, 33–88)	62.4 (15.6, 18–90)	*t* = 2.3 (182)^∧^	**0.023**
Gender (%)^*^			χ^2^ = 10.7 (1)^∞^	**0.001**
Male	56.3	32.1		
Female	43.7	67.9		
Marital status (%)^*^			χ^2^ = 4.2 (3)^∞^	0.244
Married/de facto	76.7	85.1		
Single	9.7	9.9		
Widowed	5.8	1.2		
Other	7.7	3.7		
Ethnicity (%)^*^			χ^2^ = 2.3 (3)^∞^	0.506
Caucasian	78.6	79.0		
Asian	3.9	6.2		
Middle Eastern	6.8	2.5		
Other	10.7	12.3		
Education status (%)			χ^2^ = 3.6 (3)^∞^	0.311
Tertiary	51.4	53.1		
Diploma	32.0	22.2		
High School	13.6	22.2		
Other	2.9	2.5		
Employment (%)			χ^2^ = 5.9 (2)^∞^	0.052
Working	17.5	32.1		
Retired	72.8	56.8		
Unemployed	9.7	11.1		
Support services (%)			χ^2^ = 20.9 (3)^∞^	**<0.001**
None	67.9	95.1		
Aged care package	19.4	3.7		
National disability insurance scheme	9.7	1.2		
Other	2.9	0		
Montreal Cognitive Assessment (MoCA) (SD)				
Visuospatial/executive (/5)	3.9 (1.5)	4.7 (0.7)	*t* = −4.4 (182)^∧^	**<0.001**
Naming (/3)	2.8 (0.4)	2.9 (0.2)	*t* = −1.6 (181)^∧^	0.117
Attention (/6)	4.9 (1.3)	5.4 (0.9)	*t* = −2.5 (182)^∧^	**0.012**
Language (/3)	2.4 (0.8)	2.8 (0.5)	*t* = −3.6 (182)^∧^	**<0.001**
Abstraction (/2)	1.9 (0.3)	2.0 (0.2)	*t* = −2.1 (181)^∧^	**0.036**
Delayed recall (/5)	2.8 (1.6)	3.9 (1.3)	*t* = −5.1 (182)^∧^	**<0.001**
Orientation (/6)	5.5 (0.9)	5.9 (0.3)	*t* = −3.6 (182)^∧^	**<0.001**
(MoCA) Total score (/30)	24.4 (4.8)	27.6 (2.5)	*t* = −5.4 (182)^∧^	**<0.001**
Mild cognitive impairment (<26/30) (%)	48.6	18.5	χ^2^ = 17.9 (1)^∞^	**<0.001**
Dementia (<21/30) (%)	16.5	1.2	χ^2^ = 11.9 (1)^∞^	**0.001**
36 - item short form health survey (Quality of Life Assessment) (SD)				
Physical component summary	51.6 (22.7)	79.9 (17.7)	*t* = −9.3 (182)^∧^	**<0.001**
Mental component summary	60.9 (22.2)	80.8 (17.4)	*t* = −6.6 (182)^∧^	**<0.001**
Depression characteristics^*^				
Mean Beck's depression inventory total score (SD)	11.9 (8.8)	5.2 (5.5)	*t* = 5.9 (182)^∧^	**<0.001**
Clinically depressed (%) (>13 for Parkinson's disease and >9 for caregiver groups)	38.9%	20.1%	χ^2^ = 6.8(1)^∞^	**0.009**

∧ *Independent sample t-test*.

∞ *Pearson's chi-squared test*.

* *This data is partially reproduced from Lubomski et al. (16) and Lubomski et al. (36)*.

*df, degrees of freedom; SD, Standard Deviation*.

*Bold p-values are statistically significant at p < 0.05*.

**Table 2:** Parkinson's disease clinical characteristics.

Mean age at diagnosis (years) (SD, range)^*^	58.8 (13.6, 24–88)
Mean Parkinson's disease duration (years) (SD, range)^*^	9.2 (6.5, 1–30)
Parkinson's disease phenotype (%)^*^	
Tremor dominant	30.1
Postural instability and gait impairment	20.4
Akinetic rigid	38.9
Young onset (<40 years)	10.7
Late onset (>60 years)	49.5
Disease complication (%)^*^	
Motor fluctuations	58.3
Dyskinesia	58.3
Wearing off	81.6
Impulse control disorder	19.4
Non-motor symptom (%)	
Hyposmia	73.8
REM sleep behaviour disorder	48.5
Risk factor (%)	
Family history of Parkinson's disease	18.4
Prior neuroleptic use	2.9
Any head trauma	10.7
Levodopa equivalent daily dose (mg), (SD, range)^*^	834.8 (527.3, 0–2,186)
Mean MDS unified Parkinson's disease rating scale-III (‘on’ state) (SD, range)^*^	32.9 (17.7, 5–91)
Parkinson's disease therapy (%)^*^	
Treatment naïve	4.9
Oral levodopa	89.3
Dopamine agonist	40.0
Monoamine oxidase B inhibitor	18.4
Anticholinergic	12.6
Catechol-O-methyl transferase inhibitor	23.3
Amantadine^#^	12.6
Levodopa/carbidopa intestinal gel	8.7
Deep brain stimulation	10.7
Apomorphine (subcutaneous infusion)	6.8
Quality of life	
PDQ-39 summary index (SD)	29.2 (17.3)
MDS non-motor symptoms score (NMSS) – total score (SD)	62.7 (42.9)
Gastrointestinal symptoms^*^	
Cleveland constipation score (SD)	7.2 (4.7)
Constipation score as per ROME IV criteria (SD)	4.4 (3.5)
Functional constipation as per ROME IV criteria (%)	78.6
Chronic pain over last 3 months (%)^*^	72.8
Pain score (visual analogue scale, 0–10) (SD)	4.9 (2.5)
International physical activity questionnaire (IPAQ) score (MET-minutes/week) (SD)^*^	1823.6 (1693.6)

*SD, Standard Deviation*.

# *Not considered an anticholinergic agent*.

* *This data is partially reproduced from Lubomski et al. (16)*.

### Clinical Characteristics

#### Cognitive Differences Between Parkinson's Disease Patients and Their Caregivers

PD patients showed significantly decreased cognition scores compared to their caregivers, across six out of the seven sub-scores assessed by the MoCA, with the only comparable section being “naming” (all *p* < 0.05; Table 1). The mean total MoCA score (MoCA TS) in the PD cohort was 24.4 [(SD 4.8), range 9–30], compared to the caregiver group, 27.6 [(SD 2.5), range 18–30, *p* < 0.001]. 48.6% of PD patients vs. 18.5% of caregivers met the criteria for possible MCI (*p* < 0.001), whilst 16.5% of the PD cohort met the criteria for PDD and 1.2% of the caregiver group for dementia (MoCA TS <21/30 and loss of one or more instrumental activities of daily living; Table 1) (20). This indicates that cognition is considerably lower in PD patients compared to their caregivers, accounting for disease-associated differences.

Associations between impaired cognition and a poorer QoL were identified when combining the PD patient and caregiver groups (combined cohort), with correlations between the MoCA TS and the SF-36 PCS (*r* = 0.423, *p* < 0.001) and MCS scores (*r* = 0.352, *p* < 0.001), respectively. Further, individuals in the combined cohort who were identified to have MCI, tended to have lower PCS (*r* = 0.335, *p* < 0.001) and MCS scores (*r* = 0.309, *p* < 0.001). Assessing QoL associations specifically within the caregiver group revealed correlations between the MoCA TS with the SF-36 PCS (*r* = 0.301, *p* = 0.006) and MCS scores (*r* = 0.289, *p* = 0.033). Consistently, caregivers with MCI had lower PCS (*r* = 0.277, *p* = 0.044) and MCS scores (*r* = 0.264, *p* = 0.047), signifying a lower QoL if cognitive impairment was present. Logistic regression models evaluating cognitive differences between the groups showed statistical significance for both the MoCA TS (Wald χ^2^ = 21.1, *p* < 0.001) and the proportion of individuals with MCI (Wald χ^2^ = 16.8, *p* < 0.001). Statistical significance persisted between these groups when controlling for age, sex, QoL (PCS and MCS), depression (BDI score) and constipation (Rome-IV criteria), MoCA TS (Wald χ^2^ = 3.8, df = 5, *p* = 0.044) and MCI (Wald χ^2^ = 3.7, df = 5, *p* = 0.048), respectively.

#### Cognition in Parkinson's Disease

Within the PD and caregiver cohorts, linear regression models showed a decline in cognitive function with increasing age, when controlling for sex and PD duration (β = −0.303, *r*^2^ = 0.185, *p* = 0.001 and β = −0.307, *r*^2^ = 0.101, *p* = 0.006), respectively. Significant gender differences were also identified, with male PD patients scoring a lower mean MoCA TS [23.4, (SD 5.3)] compared to PD women [25.8, (SD 3.8), *t* = −2.6, *p* = 0.011]. Linear regression modelling confirmed that men had greater cognitive impairment, corresponding to a lower MoCA TS, when controlling for age and PD duration (β = 0.274, *r*^2^ = 0.185, *p* = 0.005). A lower level of education was also associated with increased cognitive impairment, as those who completed either a diploma or high school certificate had a lower MoCA TS compared to individuals who had completed tertiary studies, independent of age, sex and PD duration (β = −0.296, *r*^2^ = 0.282, *p* = 0.002). No associations between ethnicity, marital status, employment or support service utilisation and the MoCA TS were identified. Interestingly, PD individuals with RBD were more likely to have a lower MoCA TS compared to those without RBD, after controlling for age, sex and PD duration [23.2 (SD 5.4) vs. 25.5 (SD 3.9), β = 0.186, *r*^2^ = 0.196, *p* = 0.043]. No associations between cognition and PD phenotype, dyskinesia, on/off fluctuations, ICDs, anosmia, previous neuroleptic use, head trauma, or family history of PD were identified.

#### Treatment Influences

Individuals with increased motor severity, as well as those who reported ‘wearing off’ of their treatments, were noted to have a lower MoCA TS, after controlling for age, sex, PD duration and daily LED (β = −0.475, *r*^2^ = 0.390, *p* < 0.001; β = 0.197, *r*^2^ = 0.199, *p* = 0.035, respectively). Regarding clinical management, individuals utilising anticholinergics (β = 0.195, *r*^2^ = 0.147, *p* = 0.043) and apomorphine infusions (β = 0.257, *r*^2^ = 0.226, *p* = 0.005) were more likely to have a lower MoCA TS compared to all the other standard and advanced therapies, after controlling for age, sex, and PD duration. Despite controlling for patient age and PD duration for both of these modalities, their use may select out individuals with more refractory PD and who are more prone to cognitive decline. Nevertheless, individuals with PDD were also more likely to utilise apomorphine subcutaneous infusion compared to any other standard or device assisted therapy (β = 0.199, *r*^2^ = 0.108, *p* = 0.044) and required a higher daily LED (β = −0.224, *r*^2^ = 0.106, *p* = 0.047), after controlling for age, sex, and PD duration.

#### Cognitive Impacts on Quality of Life and Non-motor Symptoms

In the PD cohort, individuals with a lower MoCA TS were noted to report a lower QoL. These scores were accompanied by correspondingly lower SF-36 PCS (β = 0.266, *r*^2^ = 0.241, *p* = 0.009) and MCS scores (β = 0.289, *r*^2^ = 0.250, *p* = 0.006), indicating increased physical and psychological disability, after controlling for age, sex and PD duration. Also, individuals who met the criteria for MCI showed poorer QoL, with lower SF-36 PCS (β = 0.203, *r*^2^ = 0.213, *p* = 0.047) and MCS scores (β = 0.325, *r*^2^ = 0.257, *p* = 0.002), when controlling for age, sex, and PD duration. QoL assessed by the PDQ-39 Summary Index (PDQ-39 SI) identified that PD patients with a lower MoCA TS (β = −0.369, *r*^2^ = 0.377, *p* < 0.001), who had MCI (β = −0.234, *r*^2^ = 0.312, *p* = 0.013) or PDD (β = −0.252, *r*^2^ = 0.326, *p* = 0.004), were more likely to report a reduced QoL (i.e., higher PDQ-39 score), after controlling for age, sex and PD duration. Perhaps also not surprisingly, individuals who reported increased NMS, assessed by the NMSS, were also more likely to show worse cognitive function, with a lower MoCA TS (β = −0.412, *r*^2^ = 0.181, *p* < 0.001) in conjunction with MCI (β = −0.324, *r*^2^ = 0.129, *p* = 0.003) or PDD (β = −0.266, *r*^2^ = 0.108, *p* = 0.009), after controlling for age, sex and PD duration.

Importantly, PD patients without MCI were noted to experience an even lower QoL when their caregiver had MCI, compared to a caregiver with no MCI [PCS 50.9 (SD 22.3) vs. 55.8 (SD 16.1), *t* = −2.5, *p* = 0.015 and MCS 60.5 (SD 18.4) vs. 64.8 (SD 19.3), *t* = −2.3, *p* = 0.028]. These findings were also observed utilising the PDQ-39 SI [28.2 (SD 23.2) vs. 25.9 (21.1), *t* = −2.1, *p* = 0.038]. No statistically significant differences in QoL change were noted for PD patients with MCI who had caregivers with or without MCI.

#### Gastrointestinal Influences

Gastrointestinal (GI) dysfunction was also associated with differences in cognitive function within the PD cohort. Individuals diagnosed with constipation according to the Rome-IV criteria, more often had MCI (β = 0.174, *r*^2^ = 0.203, *p* = 0.044), when controlling for age, sex, LED, anticholinergic medication use and PD duration. Accordingly, increasing constipation severity negatively correlated with cognitive function (i.e., lower MoCA TS) on the Rome-IV (*r* = −0.238, *p* = 0.015) and CCS (*r* = −0.240, *p* = 0.015), being most notable in individuals with PDD (Rome-IV; *r* = −0.378, *p* = 0.004 and CCS; *r* = −0.381, *p* = 0.004).

#### Mood Influences

PD patients diagnosed with depression were noted to have greater cognitive impairment (i.e., lower MoCA TS; β = −0.259, *r*^2^ = 0.251, *p* = 0.004) and were more likely to have MCI (β = −0.213, *r*^2^ = 0.222, *p* = 0.021) or PDD (β = −0.190, *r*^2^ = 0.133, *p* = 0.049), when controlling for age, sex and PD duration. The BDI score and MoCA TS showed a significant weak negative correlation (*r* = −0.284, *p* = 0.004), as well as for those individuals with a MoCA TS meeting PDD criteria (*r* = −0.226, *p* = 0.022). Furthermore, PD patients without MCI were noted to be more depressed when their caregiver had MCI, compared to a caregiver with no MCI [BDI 13.4 (SD 10.2) vs. 9.8 (SD 9.8), *t* = −1.9, *p* = 0.041]. No statistically significant changes in depression were noted for PD patients with MCI who had caregivers with or without MCI.

#### Physical Exercise and Chronic Pain Influences

PD patients who reported increased physical activity (metabolic equivalent-minutes/week, assessed by the IPAQ) were noted to have decreased cognitive impairment (i.e., higher MoCA TS; β = 0.204, *r*^2^ = 0.220, *p* = 0.040), as well as reflecting a lower likelihood of having MCI (β = 0.216, *r*^2^ = 0.217, *p* = 0.030) or PDD (β = 0.208, *r*^2^ = 0.114, *p* = 0.049), when controlling for age, sex and PD duration. Correlations between the IPAQ score and the MoCA TS (*r* = 0.251, *p* = 0.011), MCI (*r* = 0.246, *p* = 0.012) or PDD (*r* = 0.221, *p* = 0.025), were also identified. Furthermore, individuals who reported experiencing chronic pain were more likely to have increased cognitive impairment (i.e., lower MoCA TS; β = 0.187, *r*^2^ = 0.218, *p* = 0.044), after controlling for age, sex and PD duration. Interestingly, no associations between pain severity and cognitive function were identified.

## Discussion

**Figure 1:**
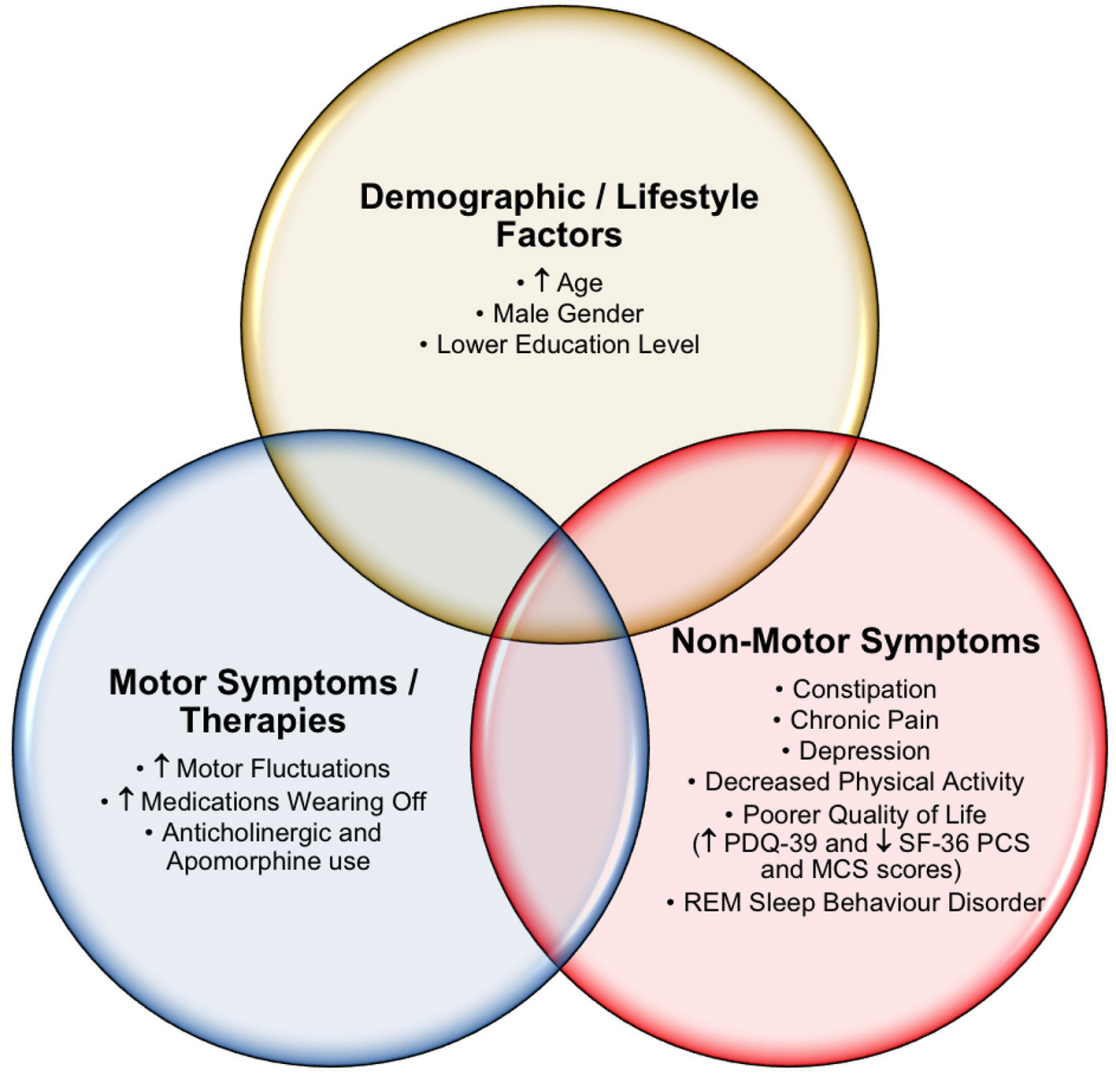
Factors associated with impaired cognition in Parkinson's disease.

Our study identified numerous clinically relevant cognitive differences across a number of demographic and clinical characteristics in our PD patient cohort (Figure 1). In addition, several important insights into PD caregivers, including the QoL and cognition of carers that may impact PD patient care, were identified. Multiple analyses were performed (PD normal cognition vs. PD MCI vs. PD caregiver with normal cognition vs. PD caregiver with MCI), with the most clinically significant results presented. Overall, PD patients showed greater cognitive impairment compared to their caregivers. Approximately half of the PD individuals and one fifth of their caregivers met the criteria for possible MCI, whilst 16.5% of PD patients met the criteria for PDD. These findings are comparably higher than in other studies (5, 20), and may be partially explained by our cohort's increased PD disease duration [mean = 9.2 years post diagnosis (SD 6.5) vs. 6.3 and ~7 years in the other studies mentioned (5, 20)].

One of the key insights of this study was the severity of cognitive impairment in PD caregivers, as this has been seldom reported in the literature. Our findings suggest that nearly a fifth of the PD caregivers in this study met the criteria for MCI, inferring a potential challenge for those with MCI being able to care for a spouse or relative with cognitive impairment. The degree of MCI detected in our caregiver group is notable, although not formally quantified in our analysis. Caregiver related cognitive impairment is important to recognise in order to provide additional support systems and services for those caring for a relative with PD. Accordingly, clinical care should not just be restricted to the PD patient but should also be a courtesy extended to the caregivers when presenting to clinic with their respective PD patient.

It was also shown that PD patients without MCI, but who had a caregiver with MCI, were more likely to report a lower QoL than those PD patients whose caregiver did not have MCI. These findings further highlight the often-complex considerations for caregiver-related MCI negatively impacting on the outcomes of PD patient management. A variety of unintentional negative outcomes may arise from PD caregivers with MCI lacking insight into appropriate caregiving responsibilities, including potentially inadequate nutrition, hygiene and medication administration for the PD patient. Furthermore, issues may arise from failure to follow through with adequate medical treatments for PD and other comorbidities, and confusion or uncertainty around when to seek additional support. Such factors may explain why PD patients with caregivers who had MCI were more likely to report a lower QoL and increased depression severity. Such considerations are vital and should prompt further studies to rigorously examine the influences of cognitive function and the degree of cognitive impairment in PD caregivers. Moreover, improved understanding of how cognitive impairment in both PD caregivers and PD patients affects the ability to provide and receive care, may further inform clinical interactions with caregivers that bolster their efforts to provide physical, emotional, functional, and financial support to relatives with PD and themselves.

Within the PD cohort, several key demographic and clinical characteristics were shown to associate with poorer cognitive function. Increasing patient age, a lower education status, the presence of RBD and male gender appeared to reflect individuals with an increased propensity for cognitive impairment. Although contradictions in the divergence of cognitive symptoms between men and women are common in the literature, men appear to perform worse on multiple measures of cognition compared to women (37–39). Perhaps unsurprisingly, advancing age (a risk factor for impaired cognition in the healthy population) and lower education levels were related to poorer cognitive capacity. Completion of tertiary studies appeared to be associated with a protective effect against cognitive decline in PD, as was reported earlier (40). Furthermore, the data indicated that RBD patients had worse cognitive impairment, which has also been described previously (41). This highlights RBD as a potential red flag for cognitive impairment in PD (41), although the exact mechanisms are still poorly understood, they are in keeping with the hypothesised caudo-rostral spread of Lewy bodies, initially through the brainstem leading to RBD and later the neocortex resulting in cognitive decline (42).

Motor severity also appeared to reflect significant associations with cognitive function in PD. Individuals with higher MDS-UPDRS-III and those reporting medications ‘wearing off’ were more likely to report a higher degree of cognitive impairment. Treatment with anticholinergics or apomorphine infusions were also associated with lower cognitive function. The following agents were considered anticholinergics; trihexyphenidyl, benztropine, orphenadrine, procyclidine or biperiden. Of the 16.5% of individuals with PDD, those requiring apomorphine infusions and a high daily LED were most vulnerable to cognitive impairment. The use of anticholinergic agents in PD is known to adversely affect cognition (43), despite another study finding no significant association in anticholinergic burden in early stage PD (44). Although apomorphine infusions are not indicated in those with severe dementia (45), it is our clinical experience that at low concentrations this adjuvant therapy can offset peak-effect dyskinesias and off-period non-motor symptoms, particularly in the older PD population who require device-assisted therapy.

When examining the PD patient and caregiver cohorts, individually and in combination, it was apparent that those individuals with more impaired cognition were more likely to report a lower QoL, indicated by the PCS and MCS scores of the SF-36. These findings are in keeping with earlier PD studies (46, 47). Individualised assessments of PD patient and caregiver QoL are vital to avoid potential oversight of the impact of QoL on PD patients that are cognitively impaired, compared to many caregivers who may not be (48), as was here. Furthermore, the impacts of impaired cognition leading to a poorer QoL have been widely studied in the PD population (47, 49). This investigation has validated these findings, supporting the association between decreased cognitive capacity and a self-perceived reduced QoL. Anticholinesterase therapy and other non-pharmacological interventions, including cognitive rehabilitation, have been proposed as useful strategies to improve attention and concentration in order to improve patient QoL (47).

Constipation has previously been identified as a potential risk factor for cognitive impairment in PD (50). However, more recently, the ascending spread of alpha-synuclein through the microbiota-gut-brain-axis has been proposed as a link between GI dysfunction and cognitive decline (51), in addition to varying metabolic pathways of the gut microbiota that affect the efficacy of PD therapies (52). Nevertheless, within our cohort, individuals with increasing constipation severity were more likely to have poorer cognitive function, when adjusted for LED and anticholinergic medication use. Important management implications arise for treating chronic constipation in PD patients with cognitive impairment, which should focus on the institution of simplified and structured regimes, incorporating extra water and added fibre intake to their diet, in addition to routine use of aperients or laxatives to minimise risk of faecal impaction. Avoidance of unnecessary anticholinergic or opioid analgesia medication or other agents that may slow gut transit times is an important consideration and clinicians should actively enquire about their PD patient's bowel motions and GI symptoms, as a potentially modifiable risk factor for worsening cognitive function.

Lastly, this study identified that the influences of depression, chronic pain and reduced physical exercise were negatively associated with cognitive function in PD. These NMS may be challenging to manage, due to interdependent relationships with executive function, which is influenced by an individual's level of motivation, engagement and insight (53–55). Complex neurotransmitter and other neuromodulatory effects are believed to be integral in the processes leading to cognitive decline in PD and may be further significantly influenced by attitudes and health-related perceptions of patients and their caregivers (56). The effects of maintaining physical exercise should not be underrated as a powerful and modifiable approach to promoting improved cognition in PD (53). Our findings support increased physical activity being positively associated with cognitive function and a reduced likelihood of having MCI or PDD. Encouragement of PD patients to remain socially active, seek help if feeling depressed or anxious, adopt depression coping strategies, maintain physical exercise, eat a healthy diet and engage in cognitive training interventions have been proposed as a multidisciplinary approach to optimising NMS and cognitive impairment in PD (36, 56–59). These interventions would be more beneficial at the MCI stage of disease, making early identification of these patients critical.

The impacts of managing many of the PD NMS that are associated with cognitive impairment also often result in notable implications for PD caregivers. Considerations include additional need for structured and timely supervision of medications, maintaining engagement and motivation with exercise regimes, seeking extra support from friends and family, as well as other support service provision options. In addition, medical and allied health consultation requirements, excess costs associated with the provision of support services, alteration to a healthy diet and medication scheduling are also often encountered. Many of these interventions are dependent on health-related perceptions and cognitive function of the PD caregivers (56). This may lead to excess caregiver stress and potential burnout (60), which can impact on the quality, standard and vigilance of care afforded to their PD patient. Therefore, impacts on caregivers need to be actively screened for and considered to ensure comprehensive and optimal clinical and home care of PD patients.

The data presented here does not explore other potential confounding factors, including anticholinesterase medication use, family history of dementia, comorbidities (including other neurological conditions) and their treatments, as well as gastrointestinal interacting medications, and are identified as limitations of this study. The anticholinergic drug burden could not be effectively calculated for either group, although several important known, as well as new, insights to cognition related differences in PD patients and their caregivers were identified. The lack of an unrelated control group with no association to PD patients may have overestimated the prevalence of cognitive impairment in the caregiver cohort, due to potential caregiver related stress caring for their spouse/family member. The results presented here should be interpreted with consideration for the limitations of the study, including self-reporting data collection, cross-sectional survey design and a potential over-representation of PD patients with cognitive impairment arising from recruitment at a single specialist PD clinic in metropolitan Sydney. The presented correlations and modelling were exploratory in nature and should be interpreted with caution. The lack of quantification of how much time caregivers spend with the PD patients was not ascertained in our study, which could reflect important implications for both the PD patient and caregiver's responses, as was the lack of a comprehensive clinical assessment of the caregivers, although data pertaining to their quality of life, gastrointestinal and depression characteristics was presented in earlier studies (16, 36, 58). Another limitation includes a small proportion of the PD cohort reporting former neuroleptic medication use, as well as any head trauma, although no statistical difference in cognitive outcomes between the two groups was seen when controlling for these factors. Further limitations include the potential confounding effects of depression severity and patient disease severity rather than disease duration when investigating treatment influences, in addition to the reliance on a single cognition screening tool, the MoCA, which may miss a large segment of single domain executive dysfunction or visuospatial dysfunction, as can been seen in PDD or Dementia with Lewy Bodies, as well as having variable sensitivity and specificity. Accordingly, a more comprehensive battery of neuropsychological assessments would be useful for characterising the full range of cognitive deficits in PD and is likely to be a more sensitive index for assessing cognitive impairment (19). Likewise, appraisal with the caregiver burden scale would be more informative in the future to explore extended impacts associated from caregiver MCI. Future studies should also assess differences in the impacts of caregiver related MCI to the care of the PD patient, by characterising if they were the sole caregiver or had other opportunities to receive support for managing complex health, financial assistance, as well as emotional well-being from other caregivers.

## Conclusion

The new insights from this study highlight the degree of MCI in PD caregivers, as well as evaluating features beyond the PD-specific factors examined in earlier studies, namely gastrointestinal dysfunction symptoms, chronic pain and physical exercise associations with PD cognitive impairment, that may positively change clinical practise. We recommend routinely screening for cognitive impairment in PD patients and suggest targeting constipation, chronic pain and promoting physical activity as part of a comprehensive model of care, which integrates multidisciplinary team involvement to achieve optimal PD patient and caregiver outcomes.

## Data Availability Statement

The data supporting the conclusions of this article can be made available by the authors upon request.

## Ethics Statement

The studies involving human participants were reviewed and approved by the Northern Sydney Local Health District Human Research Ethics Committee and the North Shore Private Hospital Ethics Committee, HREC/18/HAWKE/109, NSPHEC 2018-LNR-009, respectively. The patients/participants provided their written informed consent to participate in this study.

## Author Contributions

ML: study design, reviewed patients, collected and analysed data, drafted, and reviewed the manuscript. RD: study design, drafted, and reviewed the manuscript. CS: study design, drafted, and reviewed the manuscript. All authors contributed to the article and approved the submitted version.

## Funding

This study was completed at the Royal North Shore Hospital in Sydney Australia. It was not industry sponsored, however support was provided by a Parkinson's New South Wales, Research Seed Grant. This research was not presented at any prior meeting or conference. ML was the recipient of a RACP Research Entry and Northern Precinct Ramsay Scholarship. RD was a New South Wales Health Early-Mid Career Research Fellow. CS was a NHMRC Practitioner Fellow (APP1136800).

## Conflict of Interest

The authors declare that the research was conducted in the absence of any commercial or financial relationships that could be construed as a potential conflict of interest.

## Publisher's Note

All claims expressed in this article are solely those of the authors and do not necessarily represent those of their affiliated organizations, or those of the publisher, the editors and the reviewers. Any product that may be evaluated in this article, or claim that may be made by its manufacturer, is not guaranteed or endorsed by the publisher.
